# 763 Is Central Venous Catheter the Main Culprit for Clinically Significant Venous Thromboembolism in Burn Patients

**DOI:** 10.1093/jbcr/irae036.305

**Published:** 2024-04-17

**Authors:** Shannon Caesar-Peterson, Kaitlyn M Libraro, Abraham Houng

**Affiliations:** NY Presbyterian/ Weill Cornell Medical Center, Brooklyn, NY; New York - Presbyterian / Weill Cornell, new York, NY; NY Presbyterian/ Weill Cornell Medical College, New York, NY; NY Presbyterian/ Weill Cornell Medical Center, Brooklyn, NY; New York - Presbyterian / Weill Cornell, new York, NY; NY Presbyterian/ Weill Cornell Medical College, New York, NY; NY Presbyterian/ Weill Cornell Medical Center, Brooklyn, NY; New York - Presbyterian / Weill Cornell, new York, NY; NY Presbyterian/ Weill Cornell Medical College, New York, NY

## Abstract

**Introduction:**

Venous thromboembolism (VTE) is a significant clinical problem for burn patients. The incidence of VTE has been reported as 0.25% to 47.1%. Known risk factors include large burn size, increasing age, male gender, active smoker or alcohol use, blood transfusion and surgeries. Central venous line (CVL) use is also a known risk factor for VTE. With all the known factors, we retrospectively analyzed our patients with VTE.

**Methods:**

Retrospective chart review was performed on a single institution burn center in the calendar year of 2022. Pediatric patients were excluded. Following data were collected: age, burn size, mechanism, length of hospital stays, VTE diagnosis, VTE chemoprophylaxis, and central venous line days. VTE diagnosis was made using CT venogram or ultrasound. This project was a quality improvement initiative.

**Results:**

There were 420 patients in the study period. Of all the admitted patients, there were 6 patients diagnosed with 7 VTE. One patient had 2 separate diagnoses of VTE at 2 distinct period and anatomical site. The incidence of VTE is 1.7%. Upon further chart review, all the VTE were at the site of central venous catheters. All the patients were on VTE chemoprophylaxis. The correlation between burn size, age, length of hospital stay and VTE was not significant.

**Conclusions:**

Risk of VTE is high in burn patients due to the correlation with the Virchow’s Triad. All the patients admitted to the burn unit are placed on chemoprophylaxis unless contraindicated. The 7 patients diagnosed with VTE were on chemoprophylaxis. All the VTE were at the site of central venous catheter insertion sites, which is a known complication of catheters. The incident of 1.7% is on par with VTE incidents of burn patients, but much less than VTE caused by central venous catheters in non-burn patients. Large multicenter data collection is needed to validate this finding.

**Applicability of Research to Practice:**

Directly appliable

